# Clinical efficacy analysis of natural orifice specimen extraction surgery (NOSES) and conventional laparoscopic surgery (CLS) in the treatment of rectal cancer: a single-center retrospective analysis

**DOI:** 10.1007/s10151-025-03186-4

**Published:** 2025-07-13

**Authors:** Kai Deng, Yi-ran Li, Teng-long Guo, Jun-zhe Dou, Yu-liang Cui, Ying-feng Su

**Affiliations:** 1 Department of Gastrointestinal Surgery, Qilu Hospital of Shandong University Dezhou Hospital, Dezhou, China; 2 Department of Geriatrics (Healthcare Medicine), Qilu Hospital of Shandong University Dezhou Hospital, Dezhou, China; 3 Department of Vascular Surgery, Qilu Hospital of Shandong University Dezhou Hospital, Dezhou, China; 4 Department of Endocrinology, Qilu Hospital of Shandong University Dezhou Hospital, Dezhou, China

**Keywords:** Rectal cancer, Natural orifice specimen extraction surgery (NOSES), Conventional laparoscopic surgery (CLS), Short-term efficacy

## Abstract

**Objective:**

This study aims to compare the clinical efficacy of natural orifice specimen extraction surgery (NOSES) and conventional laparoscopic surgery (CLS) in the treatment of rectal cancer, assessing the advantages and disadvantages of both surgical approaches.

**Methods:**

A propensity score matching (PSM) method was used to analyze 221 patients with rectal cancer treated at Qilu Hospital of Shandong University Dezhou Hospital (Dezhou People’s Hospital) from January 2022 to January 2025. The NOSES group included 24 cases, while the CLS group included 197 cases. After 1:1 matching, 46 cases (23 in each group) were included. This study compared surgical time, blood loss, white blood cell count, C-reactive protein (CRP), visual analog scale (VAS) scores, time to passage of flatus, postoperative hospital stay, hospitalization costs, complications, and additional analgesia requirements between the two groups.

**Results:**

The NOSES group showed significant advantages in time to passage of flatus (1.78 ± 0.60 d versus 3.57 ± 1.08 d, *P* < 0.001), time to get out of bed (1.13 ± 0.34 d versus 1.70 ± 0.47 d, *P* < 0.001), and VAS scores on postoperative days 1 (1.70 ± 0.56 versus 3.30 ± 1.26, *P* < 0.001), 3 (1.48 ± 0.51 versus 2.91 ± 1.24, *P* < 0.001), and 7 (1.13 ± 0.55 versus 2.30 ± 1.36, *P* < 0.001) compared with the CLS group. The NOSES group also required no additional analgesia (*χ*^2^ = 9.684, *P* = 0.002). No significant differences were observed in surgical time, blood loss, or complication rates (*P* > 0.05).

**Conclusions:**

NOSES effectively alleviates postoperative pain, demonstrates significant minimally invasive advantages, and facilitates short-term patient recovery, highlighting its clinical value.

## Introduction

Colorectal cancer ranks third in global incidence and second in mortality [1]. The high incidence rate implies a significant volume of surgical treatments. With the increasing demand for improved quality of life, patients with rectal cancer seek more minimally invasive and less painful surgical options. Natural orifice specimen extraction surgery (NOSES), which allows specimen extraction through natural orifices without abdominal incisions [2], theoretically reduces postoperative pain and complications such as wound infection and incision dehiscence [3, 4]. However, owing to the specialized anastomosis techniques and complex surgical steps required, NOSES has not been widely adopted in all hospitals. This study aims to analyze the clinical efficacy of NOSES and conventional laparoscopic surgery (CLS) by comparing surgical time, blood loss, white blood cell count, C-reactive protein (CRP), visual analog scale (VAS) scores, time to passage of flatus, hospital stay, hospitalization costs, complications, and additional analgesia requirements, and provide an evidence-based foundation for surgical approach selection.

## Materials and methods

### Study population

A retrospective analysis was conducted on 221 patients with rectal cancer treated at Qilu Hospital of Shandong University Dezhou Hospital (Dezhou People’s Hospital) from January 2022 to January 2025. The NOSES group included 24 cases, and the CLS group included 197 cases.

#### Inclusion and exclusion criteria

Patients with histologically confirmed rectal cancer, tumor lower margin 4–15 cm from the dentate line, age between 18 and 80 years, and complete clinical data were included. Exclusion criteria were local invasion or distant metastasis, recurrent or multiple primary colorectal cancers, severe intestinal obstruction, perforation, or emergency surgery for bleeding, long-term opioid use, incomplete clinical and pathological data, and preoperative chemoradiotherapy.

### Ethical approval

This retrospective study was approved by the Ethics Committee of Qilu Hospital of Shandong University Dezhou Hospital (approval no. 2025016). The requirement for informed consent was waived by the Ethics Committee owing to the anonymized and de-identified nature of the data. All procedures complied with the ethical standards of the Declaration of Helsinki.

### Grouping and matching

Patients were divided into the NOSES group (*n* = 24) and the CLS group (*n* = 197) on the basis of the surgical approach. Propensity score matching (PSM) was performed using R 4.2.0 software, with age, gender, body mass index (BMI), tumor diameter, and TNM stage as covariates. These variables were selected owing to their potential influence on surgical approach selection and postoperative outcomes (e.g., age and BMI affecting surgical tolerance; gender differences in anatomical structures, pain perception, and psychosocial preferences; tumor diameter and stage determining resection extent). A caliper value of 0.05 (0.2 times the standard deviation of propensity scores) was set to balance matching precision and sample retention. Post-matching balance diagnostics, assessed via standardized mean differences (SMD) and statistical tests, revealed SMD < 10% for all covariates and no significant between-group differences (*P* > 0.05), confirming balanced baseline characteristics.

### Surgical procedures

*NOSES group*: (Figs. 1, 2, 3, 4, 5, 6, 7, 8, and 9) Surgical approaches were selected on the basis of the distance from the tumor’s lower margin to the dentate line and the Chinese Expert Consensus on NOSES for Colorectal Cancer (2023) [5].

**Fig. 1:**
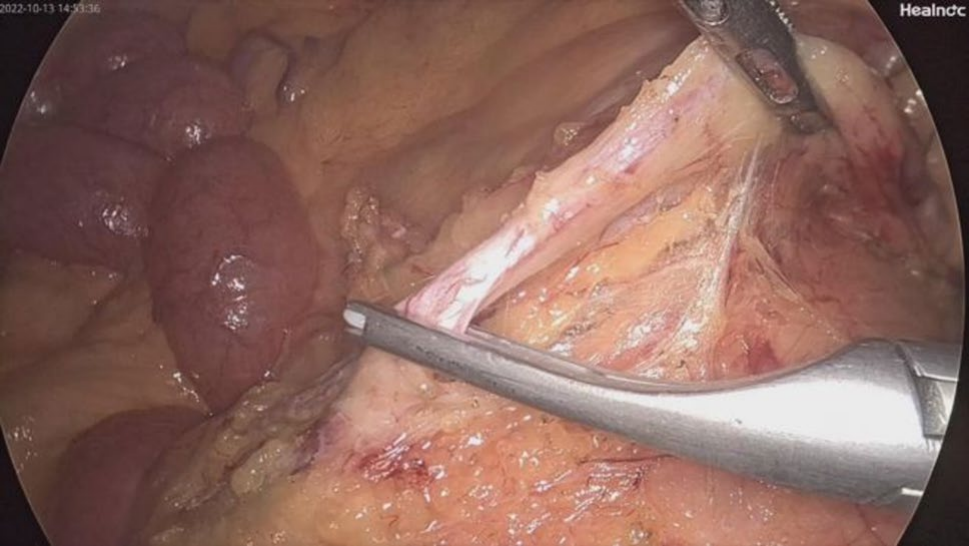
Ligation and cutting of the inferior mesenteric artery using a Hem-o-Lok clip

**Fig. 2:**
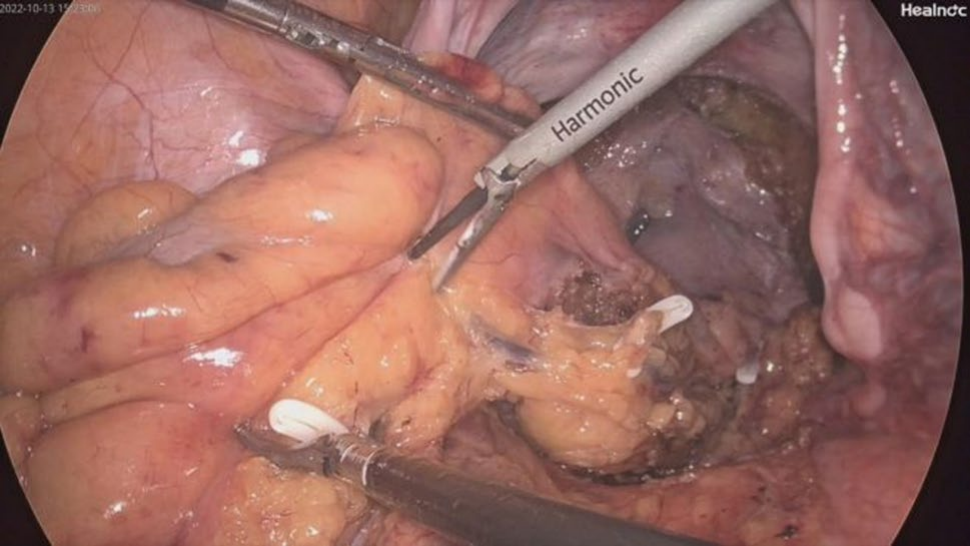
Laparoscopic dissection of the sigmoid mesocolon

**Fig. 3:**
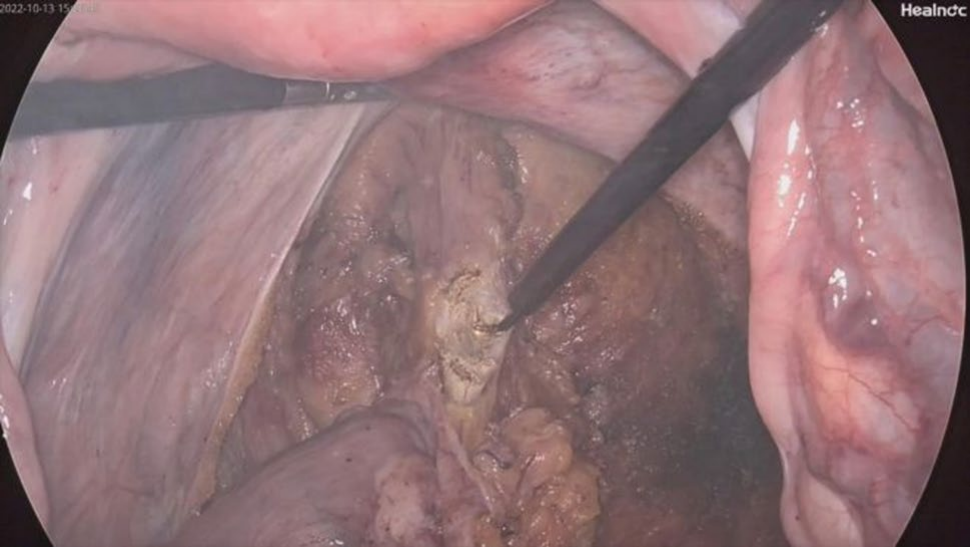
Laparoscopic incision of the distal rectal wall

**Fig. 4:**
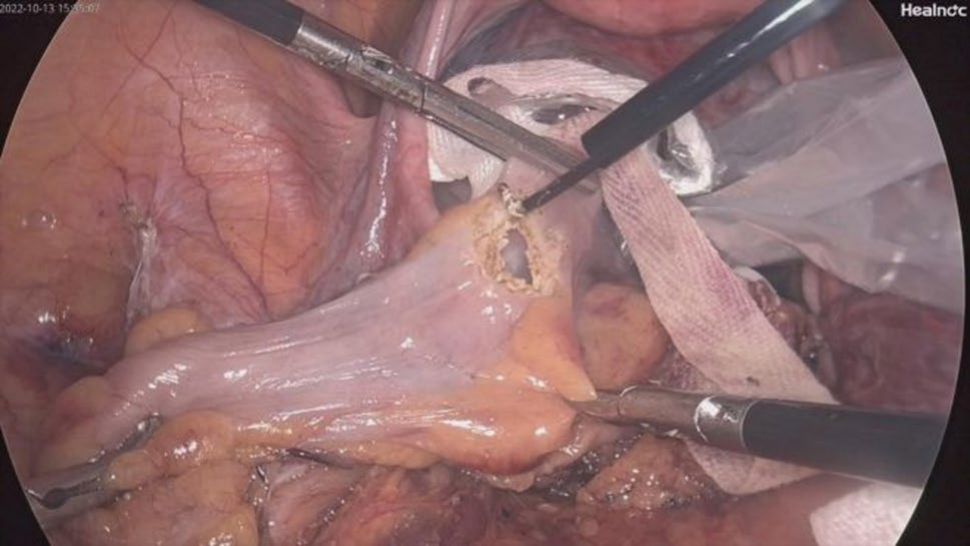
Laparoscopic incision of the sigmoid colon wall

**Fig. 5:**
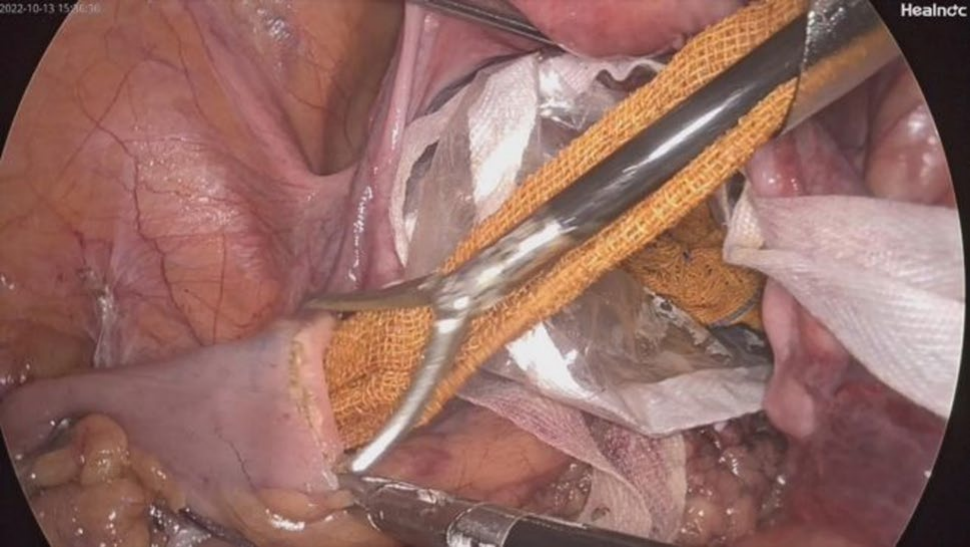
Disinfection of the colonic lumen with iodophor gauze

**Fig. 6:**
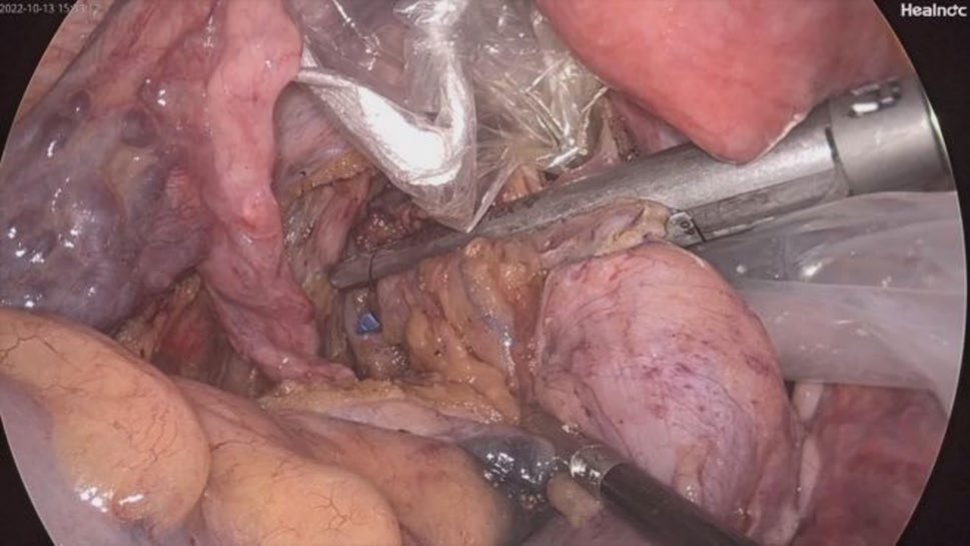
Ligation and resection of the distal rectum using the linear stapler Echelon 60

**Fig. 7:**
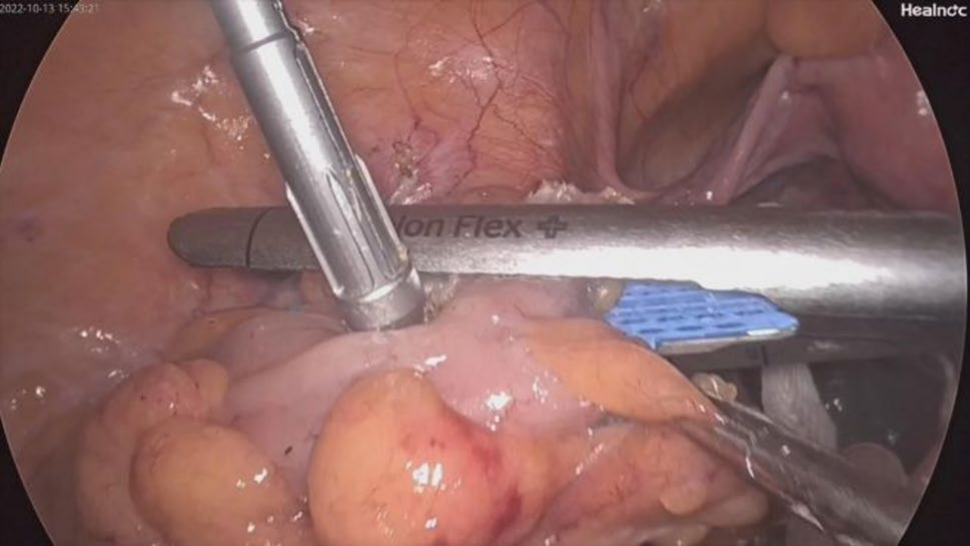
Delivery of the anvil in the sigmoid and resection of the rectum using the linear stapler Echelon 60

**Fig. 8:**
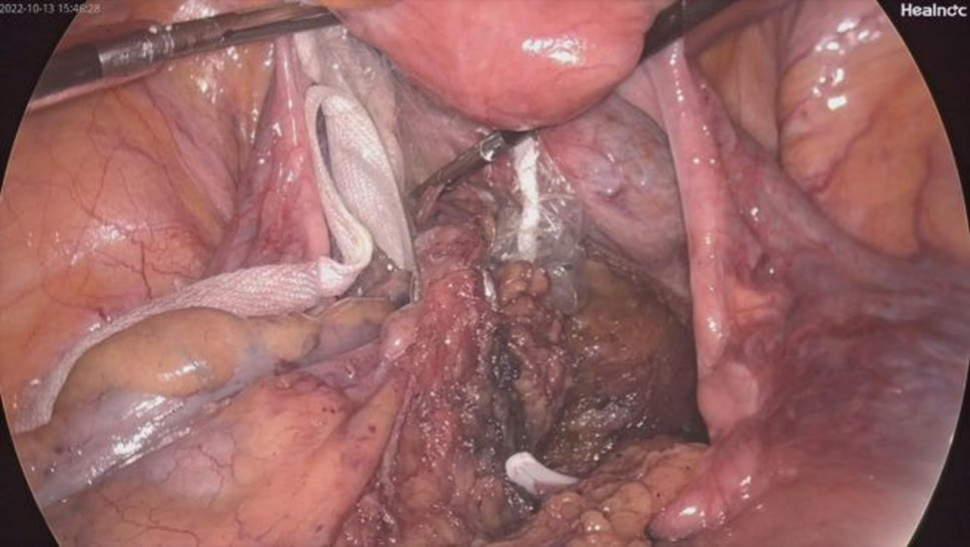
The specimen is extracted through the incision in the rectal wall and removed via the anus

**Fig. 9:**
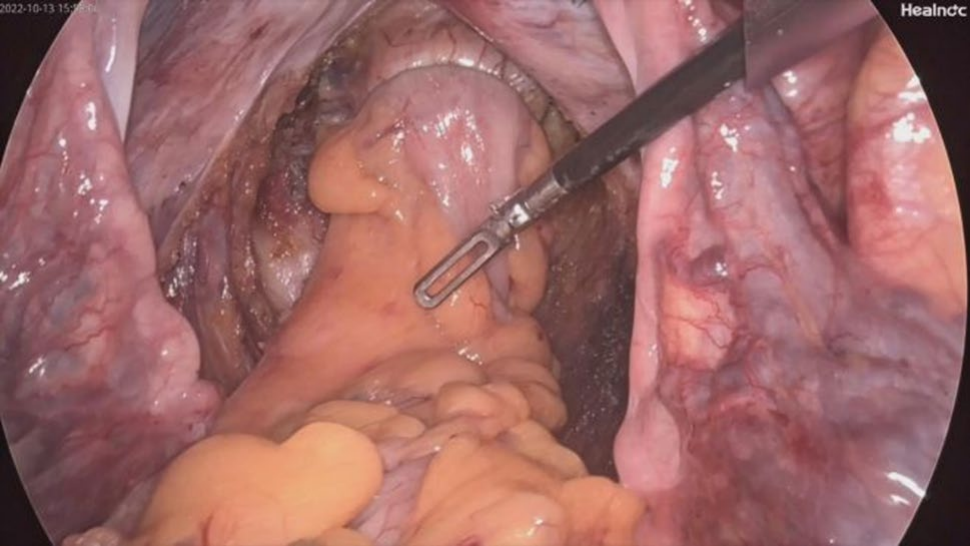
Passage of the head of the circular stapler through the anal orifice, followed by an end‐to‐end double‐stapled anastomosis

*4–5 cm*: NOSES I-type A (transanal specimen extraction with total mesorectal excision, anastomosis near the dentate line).

*5–10 cm*: NOSES II-type B (specimen extraction via rectal natural orifice, anastomosis below the peritoneal reflection).

*≥ 10 cm*: NOSES IV (specimen extraction via natural orifice, anastomosis above the peritoneal reflection).

*CLS group*: Conventional laparoscopic radical surgery was performed on the basis of tumor location: anterior resection following total mesorectal excision (TME) principles for rectal cancer. A plastic wound protector was used throughout the procedure, with specimens extracted via the ancillary incision. After dissecting the mesocolon, the proximal colon was transected, and a circular stapler anvil was placed. A temporary protective stoma was created for high-risk anastomoses.

In both groups, the bowel was mobilized via laparoscopy, and an end-to-end anastomosis was performed using a stapling device.

### Postoperative analgesia protocol

Twenty minutes prior to surgical closure, sufentanil (0.1 μg/kg) was administered intravenously for preemptive analgesia. Upon cessation of propofol and remifentanil at procedure completion, a patient-controlled intravenous analgesia (PCIA) pump was initiated. The PCIA solution contained sufentanil 150 μg and azasetron 10 mg diluted with normal saline to a total volume of 100 mL. Pump parameters were set as follows: bolus dose 4 mL, continuous infusion rate 2 mL/h, patient-controlled demand dose 2 mL, with a 5-min lockout interval. Postoperative pain management was standardized in the ward. Rescue analgesia with intramuscular ketorolac tromethamine 30 mg was administered if the visual analog scale (VAS) score remained ≥ 4 after three consecutive effective PCIA activations.

### Outcome measures

Treatment indicators were collected from the patients’ hospital records, including surgical time, blood loss, white blood cell count, C-reactive protein (CRP), visual analog scale (VAS), time to first flatus, postoperative hospital stay, hospitalization costs, postoperative complications (anastomotic leakage, intestinal obstruction, major bleeding, surgical site infection, pulmonary infection, peritonitis, urinary tract infection, urinary retention), and additional analgesia requirements. Hospitalization costs were comprehensively evaluated, encompassing: (1) surgical consumables (e.g., staplers, closure devices, ultrasonic scalpels); (2) medication expenses (parenteral nutrition, antibiotics); (3) bed and nursing fees; and (4) complication-related expenses (e.g., reoperation costs, extended antibiotic therapy). VAS was used to assess pain on postoperative days 1, 3, and 7, with higher scores indicating greater pain intensity.

### Statistical analysis

SPSS 26.0 software was used for statistical analysis. For normally distributed data (confirmed by the Shapiro–Wilk test), results were expressed as means ± standard deviations ($\overline{x}$ ± *s*), and between-group comparisons were performed using the independent samples *t*-test. Non-normally distributed data were expressed as median (P25, P75) and analyzed using the Mann–Whitney *U* test. Categorical data were presented as frequency (%) and analyzed using the *χ*^2^ test or Fisher’s exact test. Repeated measures data were analyzed using generalized estimating equations (GEE). A *P*-value of < 0.05 was considered statistically significant. No formal statistical adjustment for multiple comparisons was applied, as the study was designed to evaluate a predefined set of clinically interrelated outcomes. This approach aligns with current recommendations for surgical trials where type II error reduction is prioritized in exploratory analyses.

## Results

### Baseline characteristics

After propensity score matching (PSM), 23 patients were included in each group, with no significant differences in baseline characteristics (*P* > 0.05). After propensity score matching, no significant differences were observed between the two groups in terms of age (62.04 ± 10.07 years versus 62.74 ± 10.16 years, *P* = 0.82), gender (male: 52.17% versus 60.87%; female: 47.83% versus 39.13%, *P* = 0.55), BMI (25.00 ± 1.44 kg/m^2^ versus 24.60 ± 2.37 kg/m^2^, *P* = 0.49), tumor diameter (2.85 ± 1.25 cm versus 2.93 ± 0.93 cm, *P* = 0.80), or TNM stage (stage I: 60.87% versus 60.87%, *P* = 1.00; stage II: 34.78% versus 34.78%, *P* = 1.00; stage III: 4.35% versus 4.35%, *P* = 1.00) (Table 1).

**Table 1:** Comparison of baseline data between NOSES group and CL group before and after propensity score matching

	Before PSM			After PSM		
	NOSES	CLS	*P*	NOSES	CLS	*P*
	(*n* = 24)	(*n* = 197)		(n = 23)	(n = 23)	
Age (years)	62.02 ± 9.84	65.46 ± 9.22	0.090	62.04 ± 10.07	62.74 ± 10.16	0.817
Gender (%)			0.213			0.552
Male	12 (50)	124 (62.94)		12 (52.17)	14 (60.87)	
Female	12 (50)	73 (37.06)		11 (47.83)	9 (39.13)	
BMI (kg/m^2^)	25.50 ± 1.97	24.80 ± 1.69	0.100	25.00 ± 1.44	24.60 ± 2.37	0.498
Tumor diameter (cm)	2.80 ± 1.26	4.38 ± 1.45	< 0.001	2.85 ± 1.25	2.93 ± 0.93	0.801
TNM stage (%)			< 0.001			1
I	15 (62.5)	50 (25.4)		14 (60.87)	14 (60.87)	
II	8 (33.3)	73 (37)		8 (34.78)	8 (34.78)	
III	1 (4.2)	74 (37.6)		1 (4.35)	1 (4.35)	

### Surgical outcomes (Table 2)

**Table 2:** Intraoperative and perioperative outcomes in NOSES group and CLS group

	NOSES	CLS	*P*
	(*n* = 23)	(*n* = 23)	
Operative time (min)	165.43 ± 37.26	175.43 ± 54.08	0.469
Estimated blood loss (mL)	40.00 ± 15.67	48.70 ± 27.85	0.199
VAS score			
1 d	1.70 ± 0.56	3.30 ± 1.26	< 0.001
3 d	1.48 ± 0.51	2.91 ± 1.24	< 0.001
7 d	1.13 ± 0.55	2.30 ± 1.36	0.001
Additional analgesia required (%)	0	8 (34.78)	0.002
Time to passage of flatus (d)	1.78 ± 0.60	3.57 ± 1.08	< 0.001
Time to get out of bed (d)	1.13 ± 0.34	1.70 ± 0.47	< 0.001
Postoperative hospital stay (d)	12.52 ± 2.68	12.78 ± 5.34	0.835
WBC			
1 d	10.58 ± 2.55	9.17 ± 3.10	0.100
3 d	6.33 ± 1.56	6.39 ± 2.21	0.915
7 d	6.45 ± 2.97	6.95 ± 2.23	0.526
CRP			
1 d	43.86 ± 26.77	43.07 ± 23.89	0.917
3 d	73.28 ± 50.88	58.16 ± 31.42	0.233
7 d	39.60 ± 38.71	39.74 ± 28.06	0.989
Total hospitalization expenses (ten thousand)	5.53 ± 0.93	5.08 ± 1.31	0.180
Postoperative complications (%)	6 (26.09)	4 (17.39)	0.475
Anastomotic leakage (%)	1 (4.35)	1 (4.35)	1
Pneumonia (%)	2 (8.70)	1 (4.35)	0.550
Gastrointestinal bleeding (%)	1 (4.35)	0	0.312
Intra‐abdominal bleeding (%)	0	1 (4.35)	0.312
Intra-abdominal infection (%)	1 (4.35)	0	0.312
Gastroparesis (%)	1 (4.35)	1 (4.35)	1
Grade of morbidity (%)			0.421
Clavien–Dindo I–II	5 (21.74)	2 (8.70)	
Clavien–Dindo III–IV	1 (4.35)	2 (8.70)	

#### Surgical-related indicators

The NOSES group had slightly lower intraoperative blood loss compared with the CLS group (40.00 ± 15.67 mL versus 48.70 ± 27.85 mL, *P* = 0.19), although the difference was not statistically significant. The surgical time was similar between the two groups (165.43 ± 37.26 min versus 175.43 ± 54.08 min, *P* = 0.46).

#### Postoperative recovery indicators

**Fig. 10:**
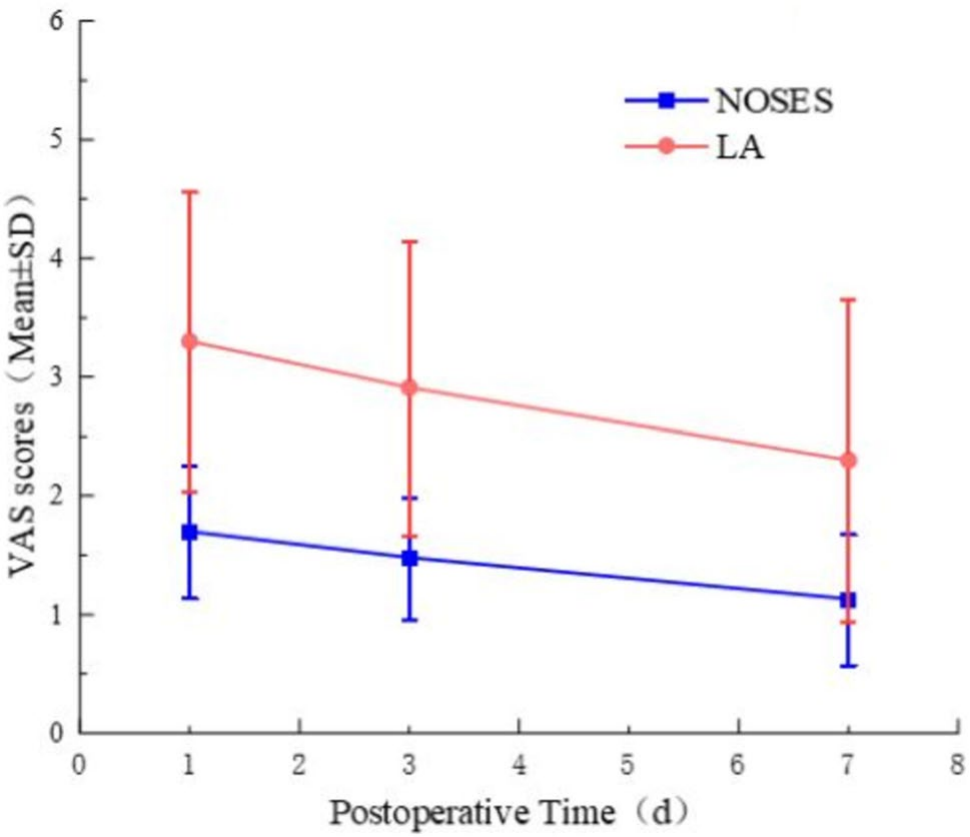
VAS: visual analog scales

The NOSES group had significantly shorter time to first flatus (1.78 ± 0.60 d versus 3.57 ± 1.08 d, *P* < 0.05) and time to get out of bed (1.13 ± 0.34 d versus 1.70 ± 0.47 d, *P* < 0.05). The visual analog scale (VAS) scores for postoperative pain were significantly lower in the NOSES group on postoperative days 1 (1.70 ± 0.56 versus 3.30 ± 1.26, *P* < 0.05), 3 (1.48 ± 0.51 versus 2.91 ± 1.24, *P* < 0.05), and 7 (1.13 ± 0.55 versus 2.30 ± 1.36, *P* < 0.05) (Fig. 10). In addition, the NOSES group required no additional analgesia (0% versus 34.78%, *P* < 0.05). There were no significant differences in postoperative hospital stay (12.52 ± 2.68 d versus 12.78 ± 5.34 d, *P* = 0.84) or total hospitalization costs (5.53 ± 0.93 ten thousand yuan versus 5.08 ± 1.31 ten thousand yuan, *P* = 0.18).

### Inflammatory response and complications

On postoperative day 1, the white blood cell count (WBC) was slightly higher in the NOSES group (10.58 ± 2.55 × 10^9^/L versus 9.17 ± 3.10 × 10^9^/L, *P* = 0.10), but no differences were observed on postoperative days 3 and 7 (*P* > 0.05). C-reactive protein (CRP) levels showed no significant differences between the groups at any time point (*P* > 0.05). All postoperative complications according to the Clavien–Dindo classification system are presented in Table 2. A total of 6 (26.09%) adverse events occurred in the NOSES group and 4 (17.39%) in the CLS group. In the NOSES group, the incidence rates of grade I–II and grade III–IV complications were 21.74% and 4.35%, respectively. In the CLS group, the rates of grade I–II and grade III–IV complications were 8.70% and 8.70%, respectively. No fatalities were recorded in either group. No significant differences were observed between the two groups in terms of the total number of complications (26.09% versus 17.39%, *P* = 0.48) and the grade of morbidity (*P* = 0.421).

### Summary of key findings

The NOSES procedure exhibited marked advantages over conventional laparoscopic surgery (CLS) in terms of postoperative pain management, gastrointestinal recovery, and early mobilization. Specifically, the NOSES group demonstrated a substantial reduction in postoperative pain, as evidenced by a 34.8–50.9% decrease in visual analog scale (VAS) scores. In addition, the time to first flatus was significantly shortened by 50.1%, and the time to get out of bed was reduced by 33.5%. These improvements were achieved without extending the surgical duration, increasing the risk of complications, or elevating medical costs. Collectively, these findings underscore the significant minimally invasive benefits of NOSES, highlighting its potential as an optimized surgical approach for rectal cancer treatment (Table 2).

## Discussion

Our study rigorously compared the short-term efficacy of natural orifice specimen extraction surgery (NOSES) and conventional laparoscopic surgery (CLS) in rectal cancer using propensity score matching (PSM). By controlling for potential confounders such as tumor stage, BMI, and demographics, we provide robust evidence supporting the minimally invasive advantages of NOSES.

NOSES avoids abdominal incisions for specimen extraction, significantly reducing abdominal wall trauma [6–8]. This advantage is particularly evident in postoperative pain control, as demonstrated by lower VAS scores in the NOSES group on postoperative days 1, 3, and 7. The reduced pain may also contribute to faster recovery, as evidenced by shorter time to first flatus and ambulation. In addition, minimizing intraoperative exposure of the bowel may reduce the risk of postoperative inflammatory bowel obstruction, promoting gastrointestinal motility and enhancing patient comfort [9–11].

Some scholars have questioned whether NOSES might violate the principles of oncological safety by potentially disseminating tumor cells during specimen extraction through natural orifices [12]. However, several studies have shown no significant differences in survival rates or oncological outcomes between NOSES and CLS [13]. For example, a prospective randomized trial found no tumor cells in peritoneal lavage fluid and no postoperative peritoneal infections in the NOSES group. A 5-year follow-up study from Beijing Shijitan Hospital also showed no significant differences in survival rates between the two groups [4]. These findings suggest that NOSES is oncologically safe [14], provided that meticulous surgical techniques and strict adherence to sterile and oncological principles are maintained [15, 16].

Despite the reduction in abdominal wall trauma, the NOSES group had a slightly higher WBC count on postoperative day 1, possibly due to minor intraoperative peritoneal contamination. However, this difference disappeared by postoperative day 3, and CRP levels remained similar between the groups. These findings suggest that NOSES does not cause significant inflammatory reactions. The overall complication rates were also similar between the groups, supporting the safety of NOSES [17–22].

Although NOSES involves more complex surgical techniques and potentially higher consumable costs, our study found no significant differences in postoperative hospital stay or total hospitalization costs [23]. This suggests that the reduced incidence of complications and shorter hospital stays may offset the higher costs of specialized equipment. The feasibility of NOSES in grassroots hospitals is further supported by cost-saving measures such as the use of suture ligation to reduce the need for stapling devices.

This study is limited by its single-center, retrospective design, small sample size, and short follow-up period. These limitations preclude a comprehensive assessment of the long-term efficacy and oncological safety of NOSES. Future studies should employ a multicenter, prospective design with long-term follow-up to further validate the clinical advantages and safety of NOSES. In addition, given the technical complexity of NOSES, its widespread adoption may be limited by the availability of experienced surgical teams and specialized training. Moreover, it is also important to note that the absence of multiplicity correction for the numerous statistical comparisons increases the probability of type I errors, particularly in secondary outcomes such as inflammatory markers. While we prioritized clinical interpretability over strict statistical adjustment, future confirmatory studies should incorporate methods such as false discovery rate (FDR) control to enhance inferential rigor.

## Conclusions

NOSES demonstrates significant minimally invasive advantages in the treatment of rectal cancer, including reduced postoperative pain and faster recovery, without increasing surgical time, medical costs, or inflammatory responses. Its oncological safety is supported by multiple studies. Therefore, NOSES has high clinical value and the potential to optimize the treatment landscape for rectal cancer. However, given its technical complexity, we recommend gradual adoption in centers with extensive laparoscopic surgical experience, along with enhanced technical training and support for grassroots hospitals.

## Data Availability

No datasets were generated or analyzed during the current study.
